# Correction: Bocci et al. Remarkable Remission Rate and Long-Term Efficacy of Upfront Metronomic Chemotherapy in Elderly and Frail Patients, with Diffuse Large B-Cell Lymphoma. *J. Clin. Med.* 2022, *11*, 7162

**DOI:** 10.3390/jcm12227053

**Published:** 2023-11-13

**Authors:** Guido Bocci, Sabrina Pelliccia, Paola Orlandi, Matteo Caridi, Marta Banchi, Gerardo Musuraca, Arianna Di Napoli, Maria Paola Bianchi, Caterina Patti, Paola Anticoli-Borza, Roberta Battistini, Ivana Casaroli, Tiziana Lanzolla, Agostino Tafuri, Maria Christina Cox

**Affiliations:** 1 Department of Clinical and Experimental Medicine, School of Medicine, University of Pisa, 56126 Pisa, Italy; 2 UOC Ematologia, Azienda Ospedaliera Universitaria Sant’Andrea, 00189 Rome, Italy; 3 Division of Hematology and Clinical Immunology, Department of Medicine, University of Perugia, 06125 Perugia, Italy; 4 Hematology Unit, Istituto Scientifico Romagnolo per lo Studio e la Cura dei Tumori (IRST) Srl—IRCCS, 47014 Meldola, Italy; 5 UOC Anatomia Patologica, Azienda Ospedaliera Universitaria Sant’Andrea & Department of Clinical and Molecular Medicine Sapienza University, 00185 Rome, Italy; 6 UOC Oncoematologia, Azienda Villa Sofia-Cervello, 90146 Palermo, Italy; 7 UOC Ematologia, Azienda Ospedaliera San Giovanni-Addolorata, 00184 Rome, Italy; 8 UOC Ematologia, Azienda Ospedaliera San Camillo, 00152 Rome, Italy; 9 Haematology Department, San Gerardo Hospital Monza, 20900 Monza, Italy; 10 UOC Medicina Nucleare, Azienda Ospedaliera Universitaria Sant’Andrea, 00189 Rome, Italy; 11 Hematology Unit, Fondazione Policlinico Tor Vergata, 00133 Rome, Italy

## 1. Error in Table Legend

In the original publication [1], there was a mistake in the legend for Table 3. The wrong term “PFS” was used instead of the right term “DFS”. The correct legend appears below.

**Table 3.** Univariate analysis for OS, EFS, and DFS of several risk factors.

## 2. Error in Table

In the original publication, there was a mistake in Table 3. The wrong term “time to progression (TTP)” was used instead of the right term “Disease-free survival (DFS)”. The corrected Table 3 appears below. 

**Table 3:** Univariate analysis for OS, EFS, and DFS of several risk factors.

Factors		EFS and OS			DFS	
	Odds Ratio	95% CI	*p* Value	Odds Ratio	95% CI	*p* Value
Hb < 12 g/dL	1.167	0.2475–5.276	1	1.125	0.2120–5.919	1
Bulky > 7.5 cm (n = 20) *	6.222	0.8219–31.78	0.0861	10	1.136–127.1	0.0635
DEVEC-light	1.429	0.2653–7.492	1	0.48	0.03458–4.368	1
No maintenance cycles following CR (n = 14)	2	0.08546–42.12	1	0	0.000–3.871	0.5055
EPI high	+∞	1.207–+∞	0.0457	+∞	0.4506–+∞	0.2725
Low albumin	0.6857	0.1427–3.442	1	0.9167	0.1423–6.078	1
Male sex	0.3673	0.07937–2.258	0.4015	0.1778	0.01380–1.408	0.179
IPI 3–5	+∞	1.207–+∞	0.0457	+∞	0.4506–+∞	0.2725
Not CR as Intermediate Response (n = 21)	6	0.7510–77.24	0.1736	+∞	1.015–+∞	0.0609
Not CR as Final Response (n = 18)	+∞	2.397–+∞	0.0049	+∞	2.397–+∞	0.0049
Super-frail	0.9643	0.1924–5.171	1	0.3667	0.02713–3.089	0.6214
Age ≥ 80	3	0.3652–42.70	0.594	0.2857	0.03816–2.418	0.2919
PS = 3	3.667	0.5689–22.33	0.3413	4.667	0.7269–26.94	0.2786

CI: Confidence interval; CR: complete response; EFS: event-free survival; EPI: elderly prognostic index; Hb: hemoglobin; IPI: international prognostic index; OS: overall survival; DFS: disease-free survival; PS: performance status. *: the number of patients for whom the factor was available is reported in parenthesis.

## 3. Error in Figure Legend

In the original publication, there was a mistake in the legend for Figure 3. The wrong term “time to progression (TTP)” was used instead of the right term “Disease-free survival (DFS)”. The correct legend appears below.

**Figure 3.** (**A**) Overall survival (OS) estimated at 24 months is 54%; (**B**) disease-free survival estimated at 24 months is 74%. The dotted black lines represent the 95% Confidence Intervals. Dots on survival curves are the events.

## 4. Error in Figure

In the original publication, there was a mistake in Figure 3. The wrong term “time to progression (TTP)” was used instead of the right term “Disease-free survival (DFS)”. No changes to the figure data have been made. The corrected Figure 3 appears below.

**Figure 3:**
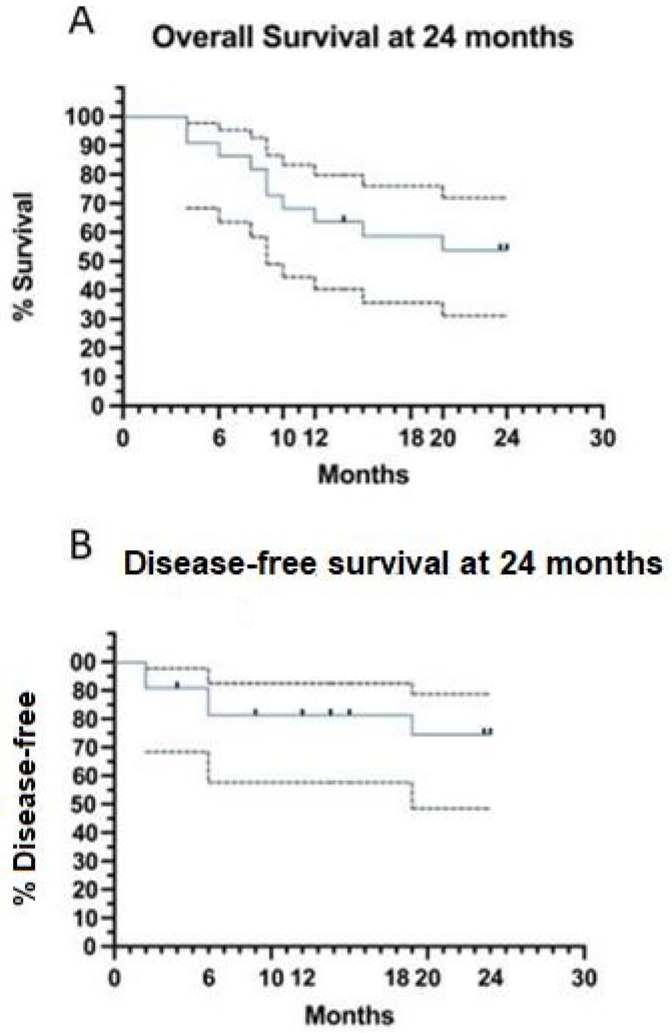
(**A**) Overall survival (OS) estimated at 24 months is 54%; (**B**) disease-free survival estimated at 24 months is 74%. The dotted black lines represent the 95% Confidence Intervals. Dots on survival curves are the events.

## 5. Text Correction

There was an error in the original publication. The wrong term “time to progression (TTP)” was used instead of the right term “Disease-free survival (DFS)” in some sections of the manuscript and the abstract. Also, a typo regarding the CI has been corrected.

A correction has been made to the Abstract section:

At the end of induction, 14/22 (64%) achieved complete remission; overall survival and event-free survival at 24 months were both 54% (95% CI = 32–72%), while the disease-free survival was 74% (95% CI = 48–88%).

A correction has been made to Results, In Vitro results:

Simultaneous exposure to metronomic VNR and ETO showed synergism (CI < 1) at effect levels exceeding 60% inhibition represented by the fraction of affected cells (Figure 1B). Synergism corresponding to CI < 1 always yielded a favourable DRI for both drugs (Table 1).

A correction has been made to Results, Clinical results:

Overall (OS) (Figure 3A) and event-free (EFS) survivals at 24 months were both 54% 95% CI = 32–72), while disease-free survival (DFS) was 74% (95% CI = 48–88%) (Figure 3B).

A correction has been made to Discussion:

In this series, the higher value of DFS versus OS reflects that a fair percentage of deaths occurred in CR patients; we would highlight that these subjects, due to their old age and burden of comorbidities, often had died for causes unrelated to DLBCL or its therapy.

The authors state that the scientific conclusions are unaffected. This correction was approved by the Academic Editor. The original publication has also been updated.

## References

[1] Bocci G., Pelliccia S., Orlandi P., Caridi M., Banchi M., Musuraca G., Di Napoli A., Bianchi M.P., Patti C., Anticoli-Borza P. (2022). Remarkable Remission Rate and Long-Term Efficacy of Upfront Metronomic Chemotherapy in Elderly and Frail Patients, with Diffuse Large B-Cell Lymphoma. J. Clin. Med..

