# Supplementary material for: Remarkable Remission Rate and Long-Term Efficacy of Upfront Metronomic Chemotherapy in Elderly and Frail Patients, with Diffuse Large B-Cell Lymphoma
Source: J Clin Med. 2022 Dec 1;11(23):7162. doi: 10.3390/jcm11237162 (PMC9739472; doi:10.3390/jcm11237162)
Supplement: Supplementary file 1 [file jcm-11-07162-s001.zip › supplementary files_revised.pptx]

## Slide 1
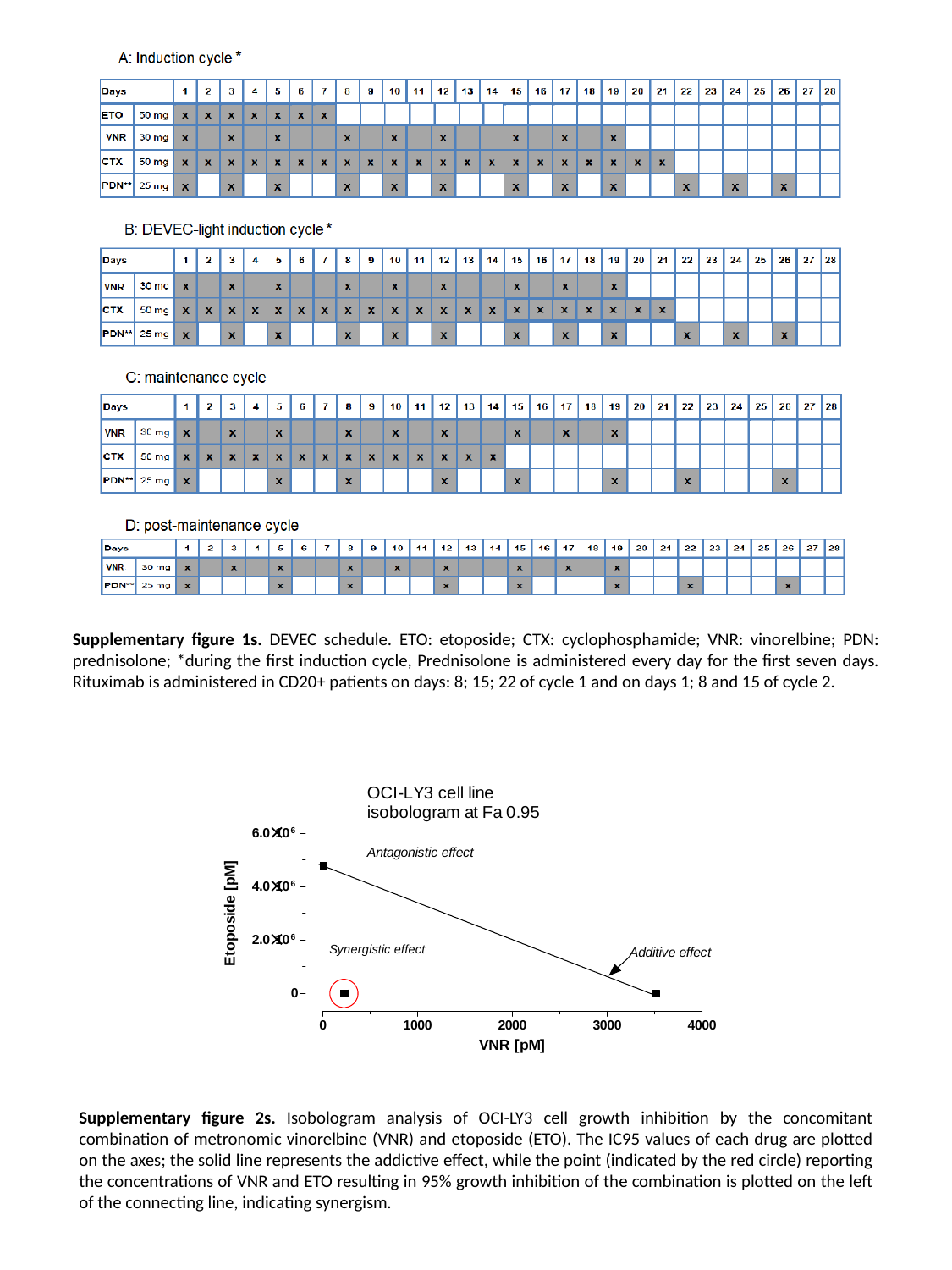

Supplementary figure 1s. DEVEC schedule. ETO: etoposide; CTX: cyclophosphamide; VNR: vinorelbine; PDN: prednisolone; *during the first induction cycle, Prednisolone is administered every day for the first seven days. Rituximab is administered in CD20+ patients on days: 8; 15; 22 of cycle 1 and on days 1; 8 and 15 of cycle 2.
Supplementary figure 2s. Isobologram analysis of OCI-LY3 cell growth inhibition by the concomitant combination of metronomic vinorelbine (VNR) and etoposide (ETO). The IC95 values of each drug are plotted on the axes; the solid line represents the addictive effect, while the point (indicated by the red circle) reporting the concentrations of VNR and ETO resulting in 95% growth inhibition of the combination is plotted on the left of the connecting line, indicating synergism.

## Slide 2
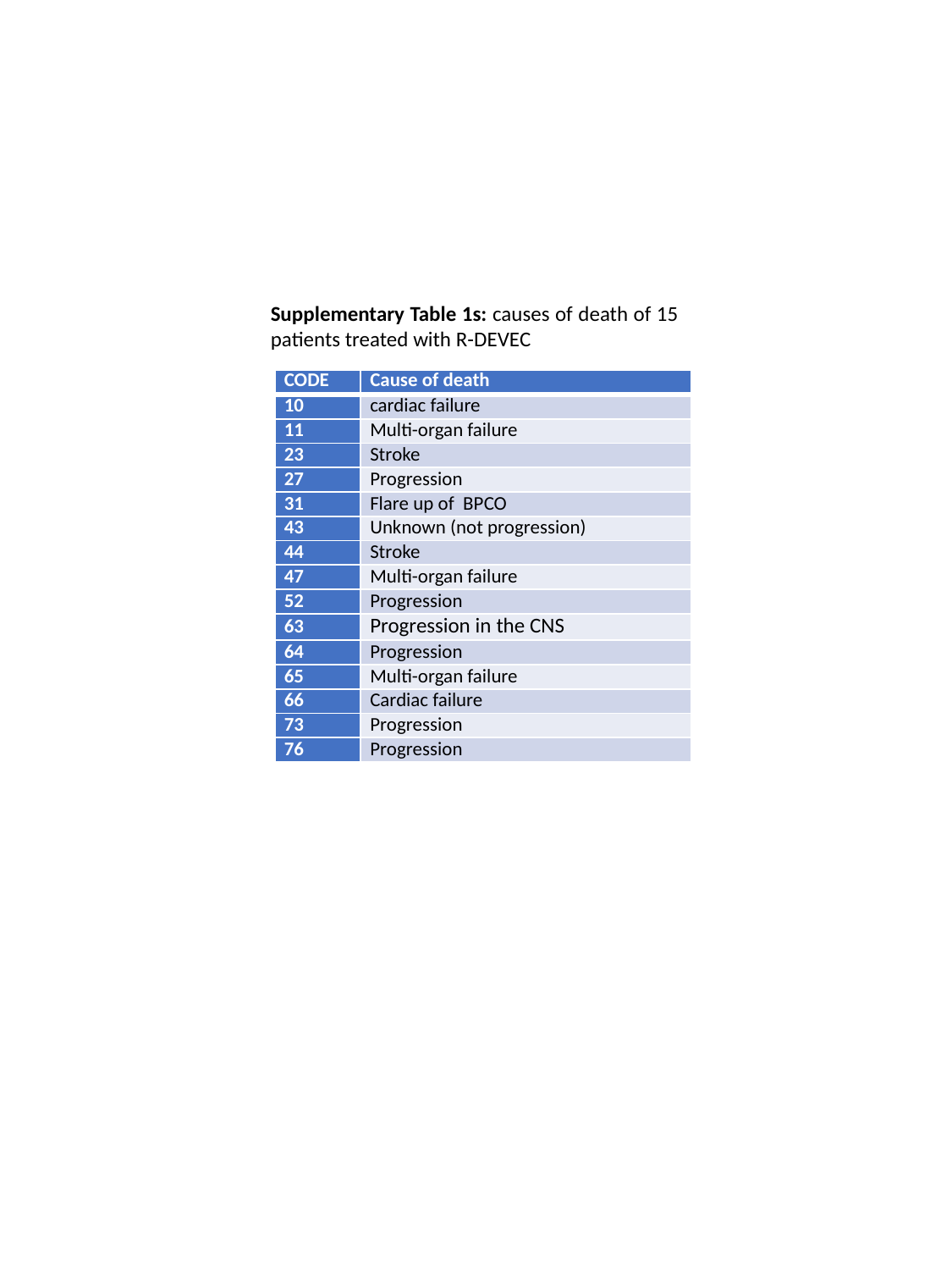

Supplementary Table 1s: causes of death of 15 patients treated with R-DEVEC
| CODE | Cause of death |
| --- | --- |
| 10 | cardiac failure |
| 11 | Multi-organ failure |
| 23 | Stroke |
| 27 | Progression |
| 31 | Flare up of BPCO |
| 43 | Unknown (not progression) |
| 44 | Stroke |
| 47 | Multi-organ failure |
| 52 | Progression |
| 63 | Progression in the CNS |
| 64 | Progression |
| 65 | Multi-organ failure |
| 66 | Cardiac failure |
| 73 | Progression |
| 76 | Progression |
